# Social Support and Breastfeeding Attitudes Among Polish Mothers of Infants and Young Children: The Mediating Role of Anxiety and Depressive Symptoms

**DOI:** 10.3390/nu18050753

**Published:** 2026-02-26

**Authors:** Aleksandra Nowicka, Agnieszka Czerwińska-Osipiak

**Affiliations:** Department of Obstetric and Gynecological Nursing, Institute of Nursing and Midwifery, Medical University of Gdańsk, M. Skłodowskiej-Curie 3 A, 80-210 Gdańsk, Poland

**Keywords:** breastfeeding, lactation, psychosocial functioning, attitude, infant, maternal health, social support

## Abstract

**Background:** Breastfeeding is essential for infant development and maternal health. Although initiation rates in Poland are high (97–99.4%), continuation of exclusive breastfeeding declines sharply to 4–22.4% at six months postpartum. In this study, the relationship between social support and attitudes toward breastfeeding was examined, focusing on the mediating role of anxiety and depressive symptoms, based on Conservation of Resources Theory. **Methods:** This cross-sectional online survey was conducted between April and October 2025. A total of 769 women aged ≥18 years with infants and children up to 24 months of age participated. Standardized tools were used: Multidimensional Scale of Perceived Social Support (MSPSS), the Iowa Infant Feeding Attitude Scale (IIFAS), and the Patient Health Questionnaire-4 (PHQ-4). Mediation analysis (PROCESS Model 4) was employed to assess indirect effects, controlling for demographic and perinatal factors. Bootstrapping (5000 samples) was implemented to determine statistical significance. **Results:** Social support was negatively correlated with anxiety–depressive symptoms (r = −0.368, *p* < 0.001) and weakly negatively correlated with breastfeeding attitudes (r = −0.075, *p* = 0.036). Anxiety–depressive symptoms showed a weak but statistically significant positive correlation with breastfeeding attitudes (r = 0.120, *p* < 0.001), which contrasts with most previous findings. Mediation analysis confirmed a significant indirect effect of social support on breastfeeding attitudes via mental health (indirect effect = −0.013, 95% CI [−0.023, −0.004]). The direct effect was non-significant (β = −0.010, *p* = 0.435). The model explained 14% of variance in anxiety–depressive symptoms and 2% in breastfeeding attitudes. **Conclusions:** Maternal mental health mediates the relationship between social support and breastfeeding attitudes. Effective lactation support should combine social support with psychoeducational interventions to reduce anxiety and depression. Integrated mental health programs in perinatal care are essential in promoting sustained breastfeeding.

## 1. Introduction

Breastfeeding is the globally recognized gold standard in infant nutrition, ensuring optimal somatic, immunological and psychomotor development of the child, as well as numerous health benefits for the mother. According to the recommendations of the World Health Organization (WHO) and other scientific societies, such as UNICEF (United Nations Children’s Fund) and AAP (American Academy of Pediatrics), exclusive breastfeeding (EBF) should be maintained for the first six months of a child’s life. Despite clear recommendations, the percentage of women exclusively breastfeeding for the suggested period remains unsatisfactory in many countries, including Poland [1,2,3,4,5,6,7,8]. In studies conducted in Poland between 2014 and 2020, it was indicated that despite a very high percentage of women initiating breastfeeding (97–99.4%), the incidence of EBF rapidly decreases in the first months of a child’s life. The percentage of infants exclusively breastfed until the age of six months varies significantly, ranging from 4% to 22.4%, depending on the study methodology, characteristics of the studied populations and data sources. Such substantial discrepancies indicate a lack of uniform, representative data on exclusive breastfeeding across the country and underscore the heterogeneity of available results [9,10,11]. This sharp decline has been attributed in studies to factors such as early return to work, limited access to professional lactation support, inconsistent institutional practices, and insufficient social and partner support [12]. Poland lacks a uniform, nationwide system for monitoring exclusive breastfeeding, and official, regularly reported statistics in this area are unavailable. The previously mentioned data are based primarily on the results of individual studies and partial analyses conducted on limited samples and specific populations, which hinders reliable assessment regarding the true scale of the phenomenon in the country. The role of a woman’s mental health during the perinatal period, particularly int the post-partum phase, including stress, anxiety and depressive symptoms, is increasingly being emphasized as a significant determinant of breastfeeding decisions.

In research to date, it is indicated that the decision to initiate and continue breastfeeding is not solely determined by medical factors, but rather is the result of a complex interaction of psychosocial ones. The most frequently analyzed factors include mothers’ level of knowledge and beliefs, their attitudes towards breastfeeding, their sense of self-efficacy, social support from their partners, family and medical staff, as well as cultural and environmental aspects. The role of a woman’s mental health during the perinatal period, particularly in the postpartum phase, including stress, anxiety and depressive symptoms, is increasingly being emphasized as a significant determinant of breastfeeding decisions [8,13,14,15].

In the international literature, mothers’ attitudes towards breastfeeding are considered a key predictor of actual breastfeeding practices, including the duration and exclusivity of breastfeeding. While attitudes reflect relatively stable beliefs and evaluations regarding breastfeeding, intentions refer to a motivational readiness to initiate or continue the behavior in a given context. Positive beliefs about health benefits, social norms and one’s own breastfeeding competencies significantly increase the likelihood of initiating and continuing breastfeeding. It is consistently indicated in research that favorable attitudes are strongly associated with longer lactation duration [16]. Mothers’ attitudes are shaped, among others, by social support, and breastfeeding self-efficacy plays a significant role. Higher self-assessment of breastfeeding competencies is associated with greater motivation to breastfeed and greater satisfaction with the process. At the same time, negative emotional states, such as increased anxiety or depressive symptoms, have been shown to indirectly influence breastfeeding practices, although the direction and nature of this relationship remain inconsistent across studies. However, sociodemographic variables (age, education, financial status) alone demonstrate a weaker and less consistent relationship with breastfeeding practices than attitudes and psychological factors, and their significance depends on cultural context [17,18,19,20].

The present study was based on Stevan E. Hobfoll’s Conservation of Resources Theory (COR), a theoretical framework that provides a key outline for explaining the relationships between psychosocial resources, stress and health behaviors. According to this theory, a fundamental human motivation is to acquire, maintain and protect resources that are valuable to oneself. Hobfoll defines resources as objects, states, personal characteristics and energies that are valued in themselves or used to achieve other valued goals. The main categories of resources include the following: object (e.g., material stability), contingent (e.g., roles and social support), personal (e.g., self-esteem, mental health), and energetic (e.g., time, energy) [21,22]. In light of the above, breastfeeding can be viewed both as a potential source of resource gain (e.g., a sense of maternal competence, closeness to the child, social approval) and as a factor in resource loss, particularly in situations of insufficient support (e.g., fatigue, frustration, perceived failure). Perceived social support is a conditional resource that protects against resource loss, facilitates coping, and increases resilience to stress. Anxiety and depressive symptoms can be interpreted as indicators of resource depletion and act as a mediating mechanism. Attitudes and intentions towards breastfeeding can be interpreted as a cognitive-affective outcome concerning the balance of resource gains and losses, as well as an expression of the subjective assessment of whether breastfeeding represents an investment in resources or a threat of resource loss for a woman [23,24].

The primary aim of this study was to analyze the relationship between perceived social support and attitudes towards breastfeeding among Polish mothers, with particular emphasis on the potential mediating role of anxiety and depressive symptoms. The secondary objective was to identify psychosocial determinants that would allow for the identification of critical areas to improve breastfeeding support strategies.

Despite numerous studies on breastfeeding, there is still a lack of comprehensive analyses in which attitudes towards breastfeeding, perceived social support and mental health indicators would be simultaneously considered in the Polish population. Most previous studies have been focused on isolated factors, omitting analysis of mediating mechanisms. This study fills this gap by offering a comprehensive approach enabling the identification of psychosocial determinants that may contribute to the initiation, continuation or premature termination of breastfeeding.

## 2. Materials and Methods

### 2.1. Study Design

This study constitutes part of the research project entitled “Health Status, Psychological and Sociodemographic Factors and Exclusive Breastfeeding in the Polish Female Population”. A cross-sectional design and an inclusive sampling approach to assess breastfeeding attitudes across a diverse population of mothers has been adopted in this research. The study participants comprised 769 mothers of infants and children up to 24 months of age. The study design was planned and described in accordance with STROBE guidelines for cross-sectional studies. All research procedures were conducted in compliance with the ethical principles outlined in the 1964 World Medical Association (WMA) Declaration of Helsinki, with subsequent amendments, which regulate research involving human participants. The study protocol was reviewed and approved by the Bioethics Committee of the Medical University of Gdańsk (decision No. KB/19/2025).

### 2.2. Study Setting

The research method used in the study was a diagnostic survey, implementing an online questionnaire that respondents completed independently. The CAWI (Computer-Assisted Web Interview) technique was employed. This approach was chosen to ensure accessibility for mothers across different regions of the country, enable nationwide reach and facilitate participation among women caring for young children. Data collection lasted from May to October 2025. Participants were recruited using convenience and snowball sampling methods via publicly available online platforms and social media groups dedicated to motherhood across Poland. This nationwide study was coordinated by researchers from the Medical University of Gdańsk, ensuring a uniform approach to data collection. To minimize selection bias, private and local groups, as well as those specifically focused on breastfeeding, were excluded to ensure the inclusion of participants having diverse preferences and experiences with infant feeding. This diversity enhances the range of perspectives captured in the study, although generalizability to the broader population of Polish mothers remains limited due to the non-random sampling method.

### 2.3. Participants

Before inclusion in the research, the selected participants received detailed information about the study’s purpose and procedure and provided informed consent electronically. Open online recruitment was used. The final analytical sample consisted of 769 mothers who met the inclusion criteria. Sociodemographic and perinatal characteristics of the study group are presented in Table 1. Variables included age, education, place of residence, financial situation, housing conditions, pregnancy and childbirth experiences, as well as results from standardized measurement tools.

### 2.4. Data Collection Tools

Data from respondents were collected using a two-part questionnaire. The first contained closed-ended questions developed by the study authors and a team of midwives, including two Certified Lactation Consultants, to best capture key sociodemographic and perinatal characteristics, which were later included in the analysis as control variables. These questions were used to address maternal age, education level, place of residence, self-assessment of financial and housing situation (each measured using a single-item, 5-point Likert-type scale ranging from ‘very bad’ to ‘very good’), number of pregnancies, mode of delivery (vaginal or cesarean section), encouragement of partner breastfeeding, occurrence of pregnancy complications and practice of uninterrupted skin-to-skin contact immediately after delivery. Brief explanations were provided to ensure consistent interpretation of clinical terms. The term “complications/illnesses before or during pregnancy” encompassed both chronic conditions and those newly developed during pregnancy (e.g., gestational hypertension, diabetes, infections). “Continuous skin-to-skin contact” referred to a standard two-hour postpartum procedure, provided that the health of the mother or child did not preclude such a procedure. Prior to the main study, this developed questionnaire was pilot-tested on a small, independent sample of mothers (*n* = 10) to confirm the clarity and comprehensibility of all items, including the clinical terms. Participants in the main study were also provided with a dedicated contact email address to seek clarification on any questions they found unclear.

Three standardized psychometric instruments were used to assess key variables, measuring subjective psychosocial aspects of maternal experience. The authors of the Polish adaptations granted permission for their use.

The Multidimensional Perceived Social Support Scale (MSPSS), adapted by Buszman and Przybyła-Basista, is used to assess the subjective sense of support available from family, friends, and a significant other. The scale contains 12 items with a seven-point Likert scale (1–7). It demonstrates very good reliability in the Polish population (Cronbach’s α > 0.90) [25,26,27].

The Iowa Infant Feeding Attitudes Questionnaire (IIFAS), adapted by Bień et al., measures attitudes towards breastfeeding. It consists of 17 statements rated on a five-point Likert scale (1–5), with nine items reverse-scored. A higher total score indicates a more positive attitude towards breastfeeding. The scale’s reliability in the Polish population is satisfactory (Cronbach’s α = 0.725) [28,29].

The Patient Health Questionnaire-4 (PHQ-4) in its Polish adaptation by Larionow and Mudło-Głagolska is a brief, validated screening tool for assessing symptoms of anxiety and depression. It comprises four items, with two items forming the anxiety subscale and two items forming the depression subscale. Responses are recorded on a four-point Likert scale (0–3). In the present study, the total PHQ-4 score was used as a continuous measure of overall anxiety–depressive symptom severity. The Polish version has demonstrated very good internal consistency, with McDonald’s ω = 0.85 for the total score [30,31].

### 2.5. Sample Size

A total of 1109 women began the survey. The inclusion criteria were maternal age ≥ 18 years and having an infant or toddler up to 24 months of age. Mothers were eligible regardless of their current or past infant feeding method (e.g., exclusive, partial or formula feeding). This inclusive approach ensured that the study captured the full spectrum of breastfeeding attitudes within a diverse population. Exclusion criteria included age < 18 years and insufficient command of Polish, which would prevent independent completion of the questionnaire. After applying these criteria and excluding questionnaires with significant missing data or ambiguous responses to control questions, the final analytical sample consisted of 769 participants.

The sample size was determined pragmatically, based on the number of eligible participants recruited during the data collection period using an open online recruitment strategy. No a priori power analysis was conducted prior to data collection. However, given the final sample size (*n* = 769), a post hoc consideration indicates that the study was adequately powered to detect small-to-moderate effects in the mediation model, including indirect effects tested with bootstrapping. For missing data not exceeding 5% of observations in individual variables, conditional mean imputation was applied, replacing missing values with the mean within relevant subgroups. This approach contributed to the preservation of the data structure and minimized the impact of missing data on variance and standard errors of the estimates.

### 2.6. Bias

Due to the online, self-report nature of the study, the potential influence of selection bias cannot be excluded. Women with higher health literacy, a particular interest in lactation or better internet access may have been more likely to participate, potentially omitting mothers with limited internet access. The attrition from 1109 starters to 769 completers may introduce a self-selection bias, potentially favoring more motivated or digitally literate participants. Recall bias is also possible, especially regarding experiences in the early postpartum period. Furthermore, some perinatal and medical variables may be subject to misclassification due to respondents’ subjective interpretation of clinical terms. There was a risk of social desirability bias—involving the provision of responses perceived as socially desirable, and declarative bias—associated with providing inaccurate information.

To minimize these biases, participants were explicitly instructed to answer all questions regarding social support and breastfeeding experiences with reference to the period when they were breastfeeding their youngest child. In addition, the study included only mothers of children up to 24 months of age, which reduces the length of the recall period. However, despite these precautions, the accuracy of retrospective self-reports may still vary depending on the child’s age at the time of survey completion. Standardized measurement tools were also used.

### 2.7. Variables

**Figure 1:**
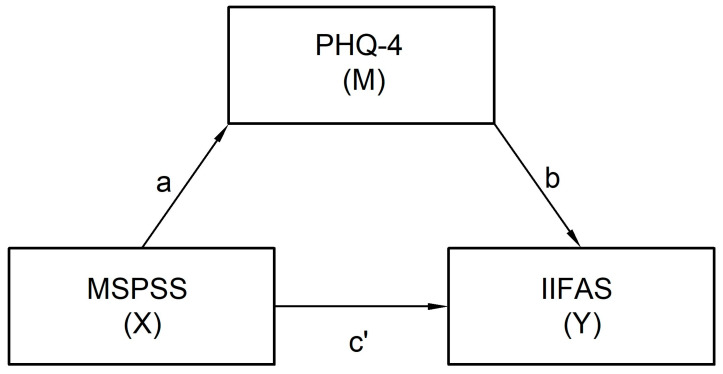
Research model of dependencies.

In the presented model, perceived social support measured using the MSPSS served as the independent variable (X), the severity of anxiety and depressive symptoms assessed using the PHQ-4 scale was treated as a mediator (M), and attitudes towards breastfeeding, measured using the IIFAS, were treated as the dependent variable (Y). The research model is presented in Figure 1.

For the model, it was assumed that the effect of the independent variable (X) on the dependent variable (Y) is mediated by variable M. This means that perceived social support influences attitudes towards breastfeeding both directly and indirectly—through its influence on the severity of anxiety and depressive symptoms.

The analyzed relationships included:

Round a (X → M), which describes the effect of perceived social support on the severity of anxiety–depressive symptoms;

Round b (M → Y), which describes the effect of anxiety–depressive symptom severity on attitudes towards breastfeeding, controlling for the effect of the independent variable;

Round c’ (X → Y), which represents the direct effect of perceived social support on attitudes towards breastfeeding after taking the mediator into account.

The indirect effect, defined as the product of rounds a and b, measured the extent to which the relationship between perceived social support and attitudes toward breastfeeding is explained by the severity of anxiety–depressive symptoms.

To limit the influence of confounding variables and increase the validity of inferences, demographic and perinatal control variables were introduced into the model. These variables, based on the literature review, may influence both mental health and breastfeeding attitudes or practices. These included maternal age, education, place of residence, self-assessment of financial situation and housing conditions, number of children, mode of delivery, partner encouragement to breastfeed, occurrence of pregnancy complications and the practice of skin-to-skin contact after delivery.

### 2.8. Statistical Analysis

Statistical analyses were performed using IBM SPSS Statistics version 29 with the PROCESS macro (Model 4). Descriptive statistics were calculated for all study variables. Categorical variables are presented as frequencies (*n*) and percentages (%), whereas continuous variables are reported as means (M) and standard deviations (SD).

The distribution of quantitative variables was assessed based on skewness and kurtosis coefficients as well as graphical inspection (histograms and normal Q–Q plots). Given the large sample size (*n* = 769 in the final analyses), formal normality tests (Shapiro–Wilk) were not relied upon due to their high sensitivity to minor deviations from normality in large samples. The absolute values of skewness (<1) and kurtosis (<2) were considered indicative of acceptable approximation to normal distribution, justifying the use of parametric statistical methods.

Pearson’s correlation coefficient (Pearson’s r) was used to assess the relationship between the study variables.

A bootstrap estimation with error correction (5000 samples) and a 95% confidence interval was used to assess the significance of the indirect effect of perceived social support (MSPSS) on breastfeeding attitudes (IIFAS) via anxiety–depressive symptoms (PHQ-4).

Prior to the mediation analysis, the assumptions of normality of residuals and absence of multicollinearity were verified. Residual plots indicated no substantial deviations from normality. Variance Inflation Factors (VIFs) for all predictors in the models were below 2.0, confirming no issues with multicollinearity.

## 3. Results

### 3.1. Bivariate Correlations Among Main Variables

To examine the basic relationships between perceived social support, anxiety–depressive symptoms and attitude towards breastfeeding, correlation coefficients were calculated. In the analysis, it was shown that social support (MSPSS) was weakly but significantly negatively correlated with attitude towards breastfeeding (IIFAS) (r = −0.075, *p* = 0.036) and moderately negatively associated with the level of anxiety–depressive symptoms (PHQ-4) (r = −0.368, *p* < 0.001). Attitude towards breastfeeding (IIFAS) showed a weak but significant positive correlation with anxiety–depressive symptoms (r = 0.120, *p* < 0.001). The results are presented in Table 2.

### 3.2. Mediation Model Testing

In mediation analysis, we examined the effect of social support (MSPSS) on attitudes towards breastfeeding (IIFAS) mediated by depressive and anxiety symptoms (PHQ-4), while controlling for several demographic and perinatal variables. In a multiple regression analysis with PHQ-4 as the dependent variable, the model was significant, explaining approximately 14% of the variance in depressive and anxiety symptoms (R^2^ = 0.143, F = 17.07, *p* < 0.001). Social support (MSPSS) showed a significant negative association with PHQ-4 (β = −0.081, *p* < 0.001), meaning that higher levels of social support were associated with lower levels of depressive and anxiety symptoms. The remaining control variables, such as maternal age, first-time mother, partner encouragement, pregnancy complications, skin-to-skin contact and vaginal delivery, did not show significant effects in this model (*p* > 0.05). The results are presented in Table 3.

In the regression analysis with the IIFAS as the dependent variable, the model was statistically significant and explained approximately 2% of the variance in the dependent variable (R^2^ = 0.024, F = 2.16, *p* = 0.028). Depressive and anxiety symptoms (PHQ-4) were a significant predictor of attitudes towards breastfeeding (β = 0.163, *p* = 0.004), whereas the direct effect of social support (MSPSS) was not significant (β = −0.010, *p* = 0.435). Maternal age showed a statistical trend toward significance in the IIFAS model (*p* = 0.069), suggesting a potential association that did not reach the conventional level of statistical significance. Other control variables, including age, first child, partner encouragement, pregnancy complications, skin-to-skin contact and vaginal delivery, did not show a significant effect on the IIFAS in this model. The results are presented in Table 4.

Analysis of the total, direct and indirect effects showed that the MSPSS influenced breastfeeding attitudes indirectly through the PHQ-4 (indirect effect = −0.013, 95% CI [−0.023, −0.004]), suggesting mediation. The remaining control variables, including age, first child, partner encouragement, pregnancy complications, skin-to-skin contact and vaginal delivery, did not show significant effects on the IIFAS in this model. The results are presented in Table 5.

In summary, the results suggest that social support influences attitudes towards breastfeeding mainly indirectly by reducing depressive and anxiety symptoms, while demographic and perinatal factors have limited significance in this model.

## 4. Discussion

The obtained results emphasize the crucial importance of mental health in the postpartum period as one of the most important areas of perinatal care [32,33,34]. The aim of this study was to determine the role of anxiety and depressive symptoms as a mediating mechanism in the relationship between perceived social support and attitudes towards breastfeeding. The analysis provided evidence of a statistically significant indirect effect consistent with mediation. The significant indirect effect, coupled with the absence of a significant direct one, confirms that the influence of social support on attitudes towards breastfeeding occurs solely through mental health resources.

### 4.1. Interpretation of Results in Light of the Conservation of Resources Theory

The lack of a direct effect of social support on attitudes towards breastfeeding is consistent with some previous research. As Leahy-Warren et al. and Schmied et al. emphasize, the importance of support depends on its quality, content and cultural context. Support can be neutral or ambivalent if it also includes acceptance of alternative forms of feeding. Furthermore, excessive pressure from the environment can lead to increased stress and a decreased sense of empowerment in women. From this perspective, social support may serve primarily an emotional function, reducing tension, rather than directly shaping health beliefs [35,36].

In relation to COR theory, social support is a classic example of a conditional resource. This means that social support does not directly influence attitudes towards breastfeeding but affects them indirectly by reducing the severity of anxiety and depression symptoms, which can be interpreted as a result of the loss or depletion of personal resources. Support can initiate a gain spiral, reducing the loss of psychological resources, which promotes more adaptive attitudes and decisions. At the same time, lack of support or its poor quality can lead to a loss spiral, manifested in increased anxiety, depressive symptoms and reduced willingness to invest in behaviors with delayed benefits, such as breastfeeding. Breastfeeding—as a practice involving a significant investment of time, energy and emotional involvement—may be perceived as an additional burden in situations of depleted resources, which is reflected in less favorable attitudes [21,22,37,38,39]. Anxiety and depressive symptoms constitute an important mechanism through which social resources influence health beliefs and attitudes. Mental health during pregnancy has been found to be a key mediator of the relationship between social support and breastfeeding attitudes. These findings highlight the central role of mental health as a determinant of the effectiveness of social support [32,40,41]. Our findings confirm this and indicate that mental health is a key mechanism linking social resources with health attitudes during pregnancy.

### 4.2. Main Findings and Comparison to Previous Studies

In the first step—the analysis stage—it was shown that a higher level of perceived social support was associated with a lower level of anxiety and depressive symptoms, a finding confirmed by both correlation analysis and multiple regression with the PHQ-4 as the dependent variable. Social support was the only significant predictor in this model, while demographic and perinatal variables such as age, number of children, partner encouragement and labor history, did not demonstrate a significant effect. This result is consistent with those obtained in numerous international studies, indicating that emotional and instrumental support from partners, family and medical personnel is a key factor in protecting women’s mental health during the perinatal period [34,42,43,44,45]. In one Polish study, however, it was demonstrated that women’s attitudes towards breastfeeding are influenced by several sociodemographic variables. These included age, marital status, financial circumstances, professional activity and having children. Women in a more stable socioeconomic situation and those who had already experienced motherhood exhibited a more positive attitude towards breastfeeding. Moreover, higher levels of life satisfaction were associated with more positive attitudes towards breastfeeding [28]. In the social context, attention is also drawn to the perception of breastfeeding in public places. In research conducted in Poland, it is indicated that although most respondents accept breastfeeding in public spaces, many women feel uncomfortable with social judgment [46]. In research conducted in the United States, Western Europe and Asia, among others, it is consistently shown that low levels of social support increase the risk of anxiety and depressive symptoms in the postpartum period, regardless of sociodemographic factors [42,43,44]. In the case of analyses conducted in China, it is noted that attitudes towards breastfeeding were significantly associated with levels of social support, knowledge about lactation and mental health, including stress. Women who received greater support from family and healthcare providers had more positive attitudes towards breastfeeding [35]. In turn, research conducted in Jordan confirmed that the determinants of attitudes towards breastfeeding include, among others, the economic situation, course of pregnancy and childbirth, previous lactation experiences and a strong intention to breastfeed [47].

The next stage of the analysis revealed that anxiety and depressive symptoms were significant predictors of attitudes towards breastfeeding, with higher levels of these symptoms associated with more positive self-reported attitudes towards breastfeeding, a counterintuitive finding discussed below. At the same time, the direct effect of social support on attitudes towards breastfeeding proved insignificant in the regression model, suggesting that the influence of support is primarily mediated through its importance for mothers’ psychological well-being. Similar results were obtained in studies conducted in various cultural contexts, in which women’s mental health, including levels of stress, depression and anxiety, was more strongly associated with breastfeeding attitudes and intentions than demographic or medical factors. For example, in trials carried out in China and Middle Eastern countries, it was shown that depressive symptoms significantly reduced positive attitudes towards breastfeeding, even with high levels of self-reported social support [47,48,49].

Mediation analysis confirmed that anxiety and depressive symptoms mediated the relationship between social support and attitudes towards breastfeeding. This indicates that support fosters more positive attitudes towards breastfeeding primarily by reducing negative psychological symptoms. This mechanism is consistent with the assumptions of the COR theory, which posits that social and psychological resources play a key role in coping with stress and making health-related decisions. In this perspective, social support serves as a resource that protects women from the loss of psychological resources, such as emotional well-being, which indirectly influences their attitudes and decisions related to breastfeeding [42,43].

It is worth emphasizing that the lack of significant influence of demographic and perinatal variables in the analyzed models indicates the limited importance of these factors in explaining attitudes towards breastfeeding in the study sample. This result may suggest that their influence is only apparent under specific psychological or cultural conditions. Similar conclusions emerge from some international studies, in which the significance of age or birth history weakened after accounting for psychological variables [44].

Recent evidence further highlights the importance of socioeconomic context in shaping breastfeeding outcomes. A systematic review by Van Neste et al. [50] demonstrated that the association between maternal socioeconomic status and breastfeeding initiation and duration differs substantially across regions. In Western Europe, higher socioeconomic status is generally associated with higher initiation rates and longer breastfeeding duration, whereas in parts of Southern Africa, this relationship may be reversed or less consistent. These findings underscore that socioeconomic influences on breastfeeding are context-dependent and shaped by broader structural, cultural, and health system factors. In light of this, the limited measurement of objective socioeconomic indicators in the present study should be interpreted with caution [50].

### 4.3. Attitudes Towards Breastfeeding, Anxiety and Depressive Symptoms

The analysis revealed a weak but significant positive correlation between positive attitudes towards breastfeeding and the severity of anxiety and depressive symptoms. Although this positive association is statistically significant, it contrasts with most previous findings reporting a negative relationship between anxiety–depressive symptoms and breastfeeding attitudes, suggesting that context-specific factors or sample characteristics may have influenced this unexpected pattern. One possible explanation is that women with a strong belief in the value of breastfeeding may experience greater emotional distress–potentially stemming from concerns about their own competence, fear of breastfeeding failure, or social pressure to meet this norm. This result is consistent with reports in which it is indicated that strong internalization of motherhood norms may paradoxically increase susceptibility to depressive and anxiety symptoms [51,52,53,54].

Pregnancy is associated with numerous biological, social and emotional changes that intensify predisposition to anxiety and depressive symptoms. According to current recommendations, the prevention of mood disorders should be an integral element of prenatal care [55]. The results of this study specify that psychological well-being plays a key role in shaping attitudes towards breastfeeding, suggesting the need to already strengthen mental health during pregnancy. At the same time, attitudes towards breastfeeding should be shaped through systematic prenatal education based on reliable knowledge, emotional support and strengthening the sense of competence of expectant mothers [56,57,58,59]. In Poland, midwives play a central role in prenatal education and lactation support. In accordance with the organizational standards of perinatal care, they care for women during pregnancy, labor and the postpartum period, providing systematic education. These activities are consistent with national recommendations for perinatal care in Poland, which emphasize structured prenatal education, breastfeeding support, and monitoring of maternal mental health [60]. Furthermore, screening for postpartum depression risks using the Edinburgh Postnatal Depression Scale (EPDS) as a standardized tool is recommended within perinatal care. The results of this study further confirm that women’s mental well-being plays a key role in shaping attitudes towards breastfeeding [61].

### 4.4. Strengths and Limitations

The strengths of this study include the use of standardized and reliable measurement tools (MSPSS, IIFAS, PHQ-4), which enhances the credibility of the obtained results. An additional advantage is the use of mediation analysis with bootstrapping, which allows for precise estimation of indirect effects. Furthermore, controlling for demographic and perinatal variables allowed for a more accurate interpretation of the results. In the context of complex perinatal decisions, even small effects can be significant, especially when they concern large populations of women.

However, significant limitations of the study should be noted. Primarily, the sample size was determined pragmatically without a priori power analysis, a methodological limitation, and the cross-sectional study design prevents causal inferences. In addition, the mediation analysis conducted on cross-sectional data cannot establish temporal precedence, and the observed associations may be influenced by potential reverse causality, where mother’s attitudes towards breastfeeding could also affect their anxiety or depressive symptoms. The use of conditional mean imputation, although justified given the very low percentage of missing values (<5%), may have led to an underestimation of variance and standard errors, potentially inflating the precision of our estimates. While we limited the sample to mothers of children up to 24 months to reduce recall bias, its potential impact remains. The accuracy of retrospective self-reports concerning psychosocial experiences from the early postpartum period may decrease over time, representing a limitation of our cross-sectional design with retrospective assessment. The use of self-report measures may be associated with response bias and the influence of socially desirable factors. Furthermore, the small effect size and low level of explained variance in the model of breastfeeding attitudes suggest that other, unaccounted-for factors, such as previous breastfeeding experience, cultural norms or the quality of prenatal education, may also influence the studied relationships.

The sample also showed an overrepresentation of women with higher education, compared to national statistics for women of reproductive age in Poland. This pattern is common in studies using open online recruitment, which tend to attract individuals with greater familiarity with digital technologies, higher health awareness, and more proactive engagement in parenting communities. Consequently, the use of convenience sampling and online recruitment may limit the external validity of the findings, as women with higher digital literacy, greater interest in breastfeeding, or better access to online platforms were more likely to participate. Therefore, the results should be interpreted with caution and cannot be directly generalized to the entire population of Polish mothers, particularly those with lower educational attainment or limited internet access. Nevertheless, this reflects current demographic trends in Poland, where the proportion of women with tertiary education continues to rise [62,63,64].

Additionally, although self-assessed financial and housing conditions were included as control variables, objective indicators of socioeconomic status (e.g., income level or employment status) were not directly measured. Given that economic context and regional disparities, as discussed in the literature, have been shown to influence both initiation and duration of breastfeeding, this represents a further limitation of the study and may have affected the observed associations.

### 4.5. Theoretical and Practical Implications

The obtained results have significant theoretical and practical implications. From a theoretical perspective, they support the application of COR theory in perinatal health research, pointing to the key role of mediating mechanisms in the relationship between social resources and health behaviors. Medical personnel, midwives and lactation consultants should pay particular attention to the mental health of pregnant women, conducting early screening for depressive and anxiety symptoms and offering appropriate forms of psychological support. These interventions can increase the effectiveness of breastfeeding education and improve women’s well-being during the perinatal period [65,66].

### 4.6. Directions for Future Research

In future research, longitudinal designs and an expanded range of variables should be incorporated to allow for the analysis of changes over time and the determination of causal relationships. Randomized controlled trials (RCTs) are also recommended to assess whether interventions that support maternal mental health can—by promoting breastfeeding—positively impact children’s metabolic programming and reduce the long-term risk of diet-related diseases in the population.

## 5. Conclusions

The influence of perceived social support on attitudes towards breastfeeding occurs only indirectly, through the severity of anxiety and depressive symptoms. Higher levels of social support are associated with lower levels of psychological symptoms, and lower levels of these symptoms promote a more positive attitude towards breastfeeding. Social support does not directly influence attitudes towards breastfeeding. Providing support alone may not be sufficient enough to improve these attitudes. Caring for the mother’s mental health is crucial. Breastfeeding support programs may be more effective if they simultaneously address anxiety and depressive symptoms, for example, through early identification of psychological risks and providing appropriate psychological support.

## Figures and Tables

**Table 1:** Study group characteristics.

Variables		M/N	SD/%
Age		32.21	4.96
Education level	Primary	2	0.3
	Vocational	9	1.2
	Secondary	121	15.7
	Higher (Bachelor’s/Master’s degree)	637	82.8
Place of residence	Village	211	27.4
	City < 100 k residents	203	26.4
	City > 100 k residents	355	46.2
Financial situation	Very good	206	26.8
	Very bad	1	0.1
	Good	450	58.5
	Satisfactory	110	14.3
	Bad	2	0.3
Housing conditions	Very good	378	49.2
	Good	312	40.6
	Satisfactory	76	9.9
	Bad	3	0.4
Parity	Primiparous	471	61.2
	Multiparous	298	38.8
Mode of delivery	Caesarean section	297	38.6
	Vaginal delivery	472	61.4
Partner’s encouragement towards breastfeeding	Not applicable	38	5.8
	No encouragement	97	12.6
	Limited encouragement	99	12.9
	Full encouragement	528	68.7
Pregnancy complications/illnesses during pregnancy	No	515	67.0
	Yes	254	33.0
Uninterrupted skin-to-skin contact during the first two hours after birth	No, it was terminated without reason	104	13.5
	No, due to maternal health	71	9.2
	No, due to infant health	85	11.1
	Yes, it was maintained	509	66.2
Iowa Infant Feeding Attitude Scale (IIFAS)		48.77	4.44
Multidimensional Scale of Perceived Social Support (MSPSS)		64.48	14.48
Patient Health Questionnaire-4 (PHQ-4)		4.04	3.19

M—mean; SD—standard deviation.

**Table 2:** Correlations between social support (MSPSS), attitude towards breastfeeding (IIFAS) and the level of anxiety and depressive symptoms (PHQ-4).

Variable	MSPSS	IIFAS	PHQ-4
MSPSS	1.000		
IIFAS	−0.075 * (*p* = 0.036)	1.000	
PHQ-4	−0.368 ** (*p* < 0.001)	0.120 ** (*p* < 0.001)	1.000

* correlation significant at the level of *p* < 0.05; ** correlation significant at the level of *p* < 0.01.

**Table 3:** Linear regression results for the PHQ-4 dependent variable.

Dependent Variable	Predictor	b	SE	t	*p*	β	Tolerance	VIF
PHQ-4	MSPSS (a)	−0.081	0.008	−10.67	<0.001	−0.368	0.997	1.003
	Age	0.010	0.024	0.41	0.679	0.014	0.881	1.136
	First child (1 = yes)	0.375	0.241	1.56	0.120	0.054	0.895	1.117
	Encouraging partner (1 = yes)	−0.441	0.328	−1.35	0.178	−0.048	0.978	1.022
	Pregnancy complications (1 = yes)	0.342	0.237	1.44	0.150	0.050	0.976	1.025
	Skin-to-skin contact (1 = yes)	−0.175	0.244	−0.72	0.473	−0.026	0.914	1.094
	Vaginal childbirth (1 = yes)	0.232	0.239	0.97	0.333	0.034	0.901	1.109
	Constant	9.066	1.028	8.82	<0.001	—	—	—
R = 0.38; R^2^ = 0.14; F = 17.07; *p* < 0.001								

**Table 4:** Linear regression results for the dependent variable, IIFAS.

Dependent Variable	Predictor	b	SE	t	*p*	β	Tolerance	VIF
IIFAS	MSPSS (c’)	−0.010	0.012	−0.78	0.435	−0.036	0.860	1.162
	PHQ-4 (b)	0.163	0.056	2.90	0.004	0.107	0.857	1.167
	Age	0.066	0.036	1.82	0.069	0.067	0.880	1.136
	First child (1 = yes)	0.461	0.362	1.27	0.204	0.046	0.892	1.121
	Encouraging partner (1 = yes)	−0.434	0.493	−0.88	0.379	−0.032	0.976	1.025
	Pregnancy complications (1 = yes)	−0.076	0.357	−0.21	0.832	−0.008	0.973	1.028
	Skin-to-skin contact (1 = yes)	0.218	0.366	0.60	0.552	0.021	0.913	1.095
	Vaginal childbirth (1 = yes)	−0.035	0.359	−0.10	0.922	−0.004	0.900	1.111
	Constan	46.695	1.626	28.72	< 0.001	—	—	—
R = 0.15; R^2^ = 0.02; F = 2.16; *p* = 0.028								

**Table 5:** Total, direct and indirect effects of perceived social support (MSPSS) on breastfeeding attitudes (IIFAS) with mediation by anxiety–depressive symptoms (PHQ-4).

Effect Type	Route	b	SE	t	*p*	95% CI	β
Total (c)	MSPSS → IIFAS	−0.023	0.012	−1.99	0.047	[−0.045; −0.000]	−0.078
Direct (c’)	MSPSS → IIFAS	−0.010	0.012	−0.78	0.435	[−0.034; 0.015]	−0.036
Indirect (a × b)	MSPSS → PHQ-4 → IIFAS	−0.013	0.005	—	—	[−0.023; −0.004]	−0.040

Note. The indirect effect was estimated using the bootstrap method (5000 samples). Models controlled for: age, first child, partner encouragement, pregnancy complications, skin-to-skin contact and vaginal delivery (0 = no, 1 = yes).

## Data Availability

The original contributions presented in the study are included in the article; further inquiries can be directed to the corresponding author.

## Abbreviations

The following abbreviations are used in this manuscript:

AAP	American Academy of Pediatrics
CAWI	Computer-Assisted Web Interview
COR	Conservation of Resources Theory
EBF	Exclusive Breastfeeding
EPDS	Edinburgh Postnatal Depression Scale
MSPSS	Multidimensional Scale of Perceived Social Support
IIFAS	Iowa Infant Feeding Attitude Scale
PHQ-4	Patient Health Questionnaire-4
STROBE	Strengthening the Reporting of Observational Biomedical Epidemiology
UNICEF	United Nations Children’s Fund
WHO	World Health Organization
WMA	World Medical Association
